# Visceral Peritoneum Hyperpigmentation in Chickens Is Associated with *DCT* Expression

**DOI:** 10.3390/ani15213076

**Published:** 2025-10-23

**Authors:** Xu Dong, Qingchun Liu, Jiabao Xing, Guodong Mo, Muyang Li, Qinghua Nie, Jingting Shu, Zhenhui Li

**Affiliations:** 1 State Key Laboratory of Swine and Poultry Breeding Industry, Guangdong Laboratory of Lingnan Modern Agriculture, South China Agricultural University, Guangzhou 510642, China; xudong@stu.scau.edu.cn (X.D.); 2774844295@stu.scau.edu.cn (Q.L.); 15267561605@163.com (M.L.); nqinghua@scau.edu.cn (Q.N.); 2 Guangdong Provincial Key Lab of Agro-Animal Genomics and Molecular Breeding, Key Laboratory of Chicken Genetics, Breeding and Reproduction, Ministry of Agriculture and Rural Affair, College of Animal Science, South China Agricultural University, Guangzhou 510642, China; 3 Key Laboratory of Zoonosis Prevention and Control of Guangdong Province, College of Veterinary Medicine, South China Agricultural University, Guangzhou 510642, China; jobsxing@stu.scau.edu.cn; 4 College of Animal Science and Technology, Guangxi Vocational University of Agriculture, Nanning 530007, China; mgd9527@163.com; 5 Key Laboratory for Poultry Genetics and Breeding of Jiangsu Province, Jiangsu Institute of Poultry Science, Yangzhou 225125, China

**Keywords:** HVP, *DCT*, melanin, melanocytes, single-cell sequence

## Abstract

**Simple Summary:**

Some chickens develop dark pigmentation inside their abdomen, known as hyperpigmentation of the visceral peritoneum. This condition lowers the visual quality of the meat, reduces its market appeal, and leads to economic losses for the poultry industry. It is especially common in certain native Chinese chicken breeds. While it is known that pigment-producing cells are involved, the exact cause of this abnormal coloring is unclear. In this study, we used advanced genetic and cellular tools to understand the condition. We discovered that the dark patches are caused by a natural pigment called melanin, and a gene named *DCT* plays a key role in producing this pigment. When this gene becomes overactive, it causes pigment cells to grow and produce more melanin than usual. We also identified a specific genetic change in *DCT* that can help the poultry industry identify birds more likely to develop this condition. Our findings provide a practical solution to reduce the occurrence of this trait through selective breeding. This can improve carcass quality, reduce waste, and increase profitability. Overall, the study helps us better understand how pigmentation works in animals and offers a clear path to improving meat quality in poultry production.

**Abstract:**

Hyperpigmentation of the visceral peritoneum (HVP) is a pigmentation abnormality in chickens that adversely affects carcass appearance, consumer acceptance, and poultry production. However, the genetic basis of HVP remains unclear. To investigate the causes and regulatory mechanisms of HVP, we employed high-performance liquid chromatography (HPLC), bulk RNA sequencing (RNA-seq), qRT-PCR, Western blotting, and siRNA interference. Additionally, single-cell RNA sequencing (scRNA-seq) was used to examine gene expression at the cellular level. Anatomical examination and hematoxylin and eosin (HE) staining revealed melanin deposition in the peritoneum of HVP-affected chickens. Spectrophotometric analysis at 500 nm showed significantly higher absorbance in the HVP group (*p* < 0.05), which correlated with the degree of pigmentation. HPLC confirmed the pigmentation as eumelanin, based on the pyrrole-2,3,5-tricarboxylic acid (PTCA) peak. RNA-seq identified 61 differentially expressed genes. Functional studies showed that dopachrome tautomerase (*DCT*) overexpression, combined with L-tyrosine (L-Tyr) supplementation, significantly increased melanin content (*p* < 0.05) and promoted melanocyte proliferation. In contrast, *DCT* silencing reduced melanin secretion and inhibited cell growth. ScRNA-seq analysis of over 9700 high-quality cells identified distinct melanocyte clusters, with *DCT* expression approximately 2.5-fold higher in melanocytes from the HVP group compared to the normal group. Furthermore, a *DCT* polymorphism (g.147917398 C > T) was identified as a potential marker for genetic selection (*p*-values = 0.033). These findings demonstrate that HVP is driven by *DCT* overexpression and excessive eumelanin deposition. *DCT* could serve as a molecular marker for genomic selection to improve poultry carcass quality and reduce economic losses in the poultry industry.

## 1. Introduction

Hyperpigmentation of the visceral peritoneum (HVP) is a quantitative trait in broiler chickens, predominantly observed in Chinese yellow-feathered breeds, particularly Huiyang Bearded Chickens [1]. Studies have demonstrated a negative genetic correlation between HVP and immune traits, such as antibody response to Newcastle disease virus (genetic correlation, rg = −0.42), suggesting that birds exhibiting HVP may have compromised immune function [1]. Additionally, HVP has been significantly associated with reduced body weight at multiple growth stages (21, 42, 70, and 91 days), indicating its broader effects on overall productivity [2]. A recent investigation reported that among 900 Huiyang Bearded Chickens at 120 days of age, approximately 37.25% exhibited visible HVP [3], further emphasizing the prevalence of this trait in commercial populations.

Although no evidence to date suggests that consumption of hyperpigmented carcasses poses health risks to humans, HVP negatively affects carcass uniformity and perceived quality, thereby diminishing market value and intensifying economic losses. As a complex trait, HVP is influenced by genetic, nutritional, physiological, environmental, and pharmacological factors [4,5]. However, moderate to high heritability estimates (h^2^ = 0.33–0.452) suggest that genetic factors play a dominant role in determining HVP expression [1,2].

Melanin, the pigment responsible for coloration in animals, is produced by melanocytes and exists primarily in two forms: eumelanin and pheomelanin [6,7]. Pigment cells are known to influence skin and feather coloration, and disturbances in melanin regulation have been linked to pigmentation disorders [8]. In birds, the genetics and physiology of melanin synthesis have been well studied in skin and feather pigmentation. A well-known example of extreme hyperpigmentation is found in Silkie chickens [9,10], which exhibit fibromelanosis (FM), a phenotype driven by the endothelin 3 (*EDN3*) locus and other regulatory elements, including the sex-linked inhibitor of dermal melanin (ID), inherited in a Mendelian semi-dominant manner [11,12,13]. Histological studies have identified melanosome-like granules in the visceral peritoneum of chickens affected by HVP [14,15,16,17,18], indicating active melanin biosynthesis at this site.

In response to these gaps in knowledge and the urgent need to reduce HVP incidence in poultry production, this study employed an integrative multi-omics approach. By combining bulk RNA sequencing (RNA-seq), single-cell RNA sequencing (scRNA-seq), and molecular validation, we identified eumelanin accumulation as the primary cause of HVP in Huiyang Bearded Chickens. Our results demonstrate that differential expression of the dopachrome tautomerase (*DCT*) gene plays a key regulatory role in HVP development. These findings not only advance our understanding of non-dermal melanogenesis in birds but also identify a specific SNP locus significantly associated with HVP traits, the *DCT* polymorphism (g.147917398 C > T, as detailed in Supplementary File S1), which provides valuable molecular targets for selective breeding strategies aimed at improving carcass quality and production efficiency.

## 2. Materials and Methods

### 2.1. Ethics Statement

All experimental animal procedures were approved by the Institutional Animal Protection and Utilization Committee of South China Agricultural University (approval ID: 2023g021, approval date: 21 November 2023). The animals involved in the study were treated humanely.

### 2.2. Experimental Animals

All animals used in this study were purchased from Huizhou Longmen Sanhuang Chicken Breeding Co. (Huizhou, China). A total of 60 female Huiyang Bearded chickens, aged 40 days, were randomly selected for slaughter and tissue sampling. Prior to slaughter, the chickens were humanely stunned using an electroshock bath to ensure they were unconscious. They were then euthanized by severing the jugular vein and carotid artery on one side of the neck, ensuring a quick and painless death. After slaughter, the visceral peritoneum was collected for phenotypic evaluation, with samples placed on ice. Three samples each from normal peritoneum and hyperpigmented peritoneum were preserved in 4% paraformaldehyde. The remaining peritoneal tissues, along with other collected tissues such as skin, internal organs, and muscle, were frozen in liquid nitrogen and stored at −80 °C for further analysis.

Additionally, 347 female Huiyang Bearded chickens, aged 40 days and from the same batch, were selected for live phenotypic characterization of the visceral peritoneum. Among these, 162 chickens exhibited normal peritoneum, while 185 showed hyperpigmented peritoneum. Blood samples were collected via venipuncture from each chicken, and DNA was extracted for Sanger sequencing to provide genotypic data for SNP loci analysis.

For primary melanocyte isolation, Silkie chicken eggs at 20 days of embryonic development were used. In addition, three 40-day-old female Silkie chickens were slaughtered to separate the peritoneum and used as the positive control group in the detection of melanin content.

### 2.3. Hematoxylin and Eosin Staining

The fresh normal peritoneum and hyperpigmented visceral peritoneum tissues were fixed in 4% paraformaldehyde at 4 °C for 24 h, and then dehydration and paraffin embedding were carried out by the gradient alcohol method as follows: Place the dehydration cassette into a dehydration machine and proceed with a sequential gradient dehydration using alcohol solutions. This involves a 4 h incubation in 75% alcohol, followed by 2 h in 85% alcohol, 2 h in 90% alcohol, 1 h in 95% alcohol, a 30 min immersion in absolute ethanol I, another 30 min immersion in absolute ethanol II, a 5 to 10 min immersion in alcohol–benzene, two 5 to 10 min immersions in xylene I and xylene II, respectively, and finally, a 1 h incubation at 65 °C for each of paraffin I, paraffin II, and paraffin III. After trimming, place the wax-soaked tissue in a −20 °C cryostat for cooling. Subsequently, position the cooled wax block in a paraffin microtome for slicing at a thickness of 4 μm. The tissue sections will float in a water bath at 40 °C to flatten them. Lift the tissue sections onto glass slides and then bake them in a 60 °C oven. After the water and wax have dried, remove the sections and store them at room temperature for staining.

### 2.4. High-Performance Liquid Chromatography Detection

For the preparation of peritoneal samples and melanin standards, the crushed bearded chicken peritoneum was measured. Specifically, phosphate buffer and 2% by mass of papain and trypsin were added based on the sample’s mass (3 mL of phosphate buffer and the corresponding amount of enzyme per 1 g of sample). The mixture was then stirred for enzymatic digestion at 55 °C for 24 h, followed by centrifugation. The supernatant was discarded, and the precipitate was rinsed multiple times with petroleum ether, anhydrous ethanol, and distilled water. Vacuum drying was performed to obtain crude melanin from the bearded chicken peritoneum.

The crude melanin was dissolved in 10 mL of 1 mol/L K_2_CO_3_ solution, to which 0.5 mL of 30% H_2_O_2_ was added, and the reaction was carried out in boiling water for 20 min. The solution was then cooled under running water, and the reaction was terminated by adding 0.6 mL of 10% Na_2_SO_3_ solution. The pH was adjusted to 1.0 by adding 6 mol/L HCl. Extraction with 40 mL of ether was performed, retaining the organic phase, and repeating the process twice. The solution was left in a fume hood overnight. It was later dissolved and passed through a 0.45 μm organic filter membrane to obtain the sample solution for testing.

The chromatography conditions were as follows: chromatographic column: LAEQ-462571 Athena C18 liquid chromatographic column (4.6 × 250 mm, 5 μm); mobile phase: 0.1% chromatographic-grade formic acid: chromatographic-grade methanol = 6:4; elution rate: 0.6 mL/min; elution temperature: 30 °C; detection wavelength: 269.8 nm; injection volume: 20 μL each time.

### 2.5. Spectrophotometric Detection

The peritoneum was ground into a fine powder and then underwent vacuum freeze-drying to produce a dry substance. An exact amount of 0.1 g of the sample was blended with 1 mL of Soluene-350 and ddH2O in a 9:1 (*v/v*) ratio. This mixture was sealed and submerged in a boiling water bath at 100 °C. Once the solution achieved a complete yellow coloration, it was promptly extracted and inserted into a Nanodrop 2000 spectrophotometer (Thermo Fisher Scientific, Wilmington, DE, USA) to determine the absorbance value at 500 nm (A500). The melanin content of the tissue samples was subsequently compared based on the magnitude of absorbance.

### 2.6. Total RNA Extraction and RNA-Seq

The total RNA was extracted from both the HVP sample and the normal sample using Trizol Reagent (Invitrogen, Carlsbad, CA, USA) in accordance with the manufacturer’s recommended protocol. Subsequently, the integrity of the RNA was assessed through agarose gel electrophoresis, while the RNA concentration and purity were determined using a NanoDrop 2100 (Thermo Fisher Scientific, Fremont, CA, USA). High-quality RNA obtained from this process was utilized in the construction and sequencing of transcriptome libraries. To elaborate further, the total RNA transcriptomic libraries were constructed employing the TruSeqTM Stranded Total RNA Library Prep Kit (Illumina, San Diego, CA, USA) for Illumina sequencing. The samples were subsequently subjected to sequencing on the Illumina HiSeq platform with PE150 paired reads. To ensure data quality, the resulting fastq reads were scrutinized using FastQC v0.11.9 (Babraham Bioinformatics, Cambridge, UK). Subsequently, they were aligned to the reference genome using Bowtie2 v2.4.2 with default settings. The generated SAM files were then converted to BAM format using SAMtools 1.11. For transcriptomic analysis, Feature Counts 2.0.1 was employed to acquire the raw gene counts.

### 2.7. Tissue Dissociation and Preparation of Single-Cell Suspensions

In this experiment, we selected a total of 20 Huiyang Bearded chickens, all 40 days old, including 10 with normal peritoneum and 10 with hyperpigmented visceral peritoneum. All procedures strictly followed ethical guidelines for animal welfare. Prior to slaughter, the chickens were humanely stunned using an electrical water bath to ensure unconsciousness, followed by exsanguination through severing the jugular vein and carotid artery on one side of the neck to ensure a quick and painless death. Subsequently, the peritoneal tissues were promptly excised using surgical scissors. The excised tissues underwent immediate rinsing, being submerged 2–3 times in pre-chilled 1 × PBS maintained at 4 degrees Celsius on ice. They were then transferred to 1.5 mL centrifuge tubes. Next, the tissues were finely minced into small pieces, approximately 0.5 mm^2^ in size, using surgical scissors. These mince specimens were incubated for 30 min on a constant-temperature shaker at 37 °C, utilizing a solution of 0.1 mg/mL collagenase 1 (Invitrogen). The termination of the digestion process was initiated as soon as the digestive solution turned turbid and the tissue mass dissolved. This was achieved by introducing a complete culture (Dulbecco’s modified Eagle medium, DMEM) containing 10% fetal bovine serum (Gibco, Carlsbad, CA, USA). The resulting suspension was subsequently filtered through a 40 µm cell sieve, and the cells were collected through centrifugation (5 min at 1000 rpm, 4 °C). After discarding the supernatant, a 1 mL re-suspension solution (pre-chilled PBS containing 1% bovine serum albumin) was introduced into the centrifuge tube to re-suspend the cells. Subsequently, 3 mL of pre-chilled erythrocyte lysis solution (Sorvall) was added and evenly mixed through pipetting.

Following a 5 min incubation at 4 °C, the mixture underwent immediate centrifugation at 4 °C (1000 rpm) for an additional 5 min. The supernatant was discarded once more, and a 1 mL re-suspension solution (pre-chilled 1 × PBS) was added to achieve full cell re-suspension. Cell quality was evaluated using a cell counter, with cell concentration being adjusted to a range of 700–1200 cells/μL based on the quality assessment results. Cell viability was assessed using AO/PI dual fluorescent dye (Countstar Rigel S2) to ensure that cell activity exceeded 90%, with agglomeration being less than 15%.

Once the final cell concentration and activity met the specified criteria, the cells were maintained on ice and used in a single-cell transcriptome chip-on-board experiment within 30 min, utilizing the 10× Genomics platform.

### 2.8. Single-Cell RNA Sequence Library Preparation and Sequencing

In compliance with the manufacturer’s instructions provided for the Chromium Next GEM Single Cell 3’ Reagent Kit v3.1 (10× Genomics, Pleasanton, CA, USA), we proceeded with the construction of single-cell sequencing libraries. To elaborate on the process, the cell suspension was loaded onto the Chromium Next GEM Chip G, and the Chromium Controller was operated to generate single-cell gel beads in emulsions (GEMs), as per the manufacturer’s recommendations. The captured cells were lysed, and the released RNA was barcoded through reverse transcription within individual GEMs. This procedure resulted in the production of barcoded, full-length cDNA, and the subsequent library construction adhered to the manufacturer’s protocol.

Subsequently, the Qubit 4.0 was employed to measure the concentration of cDNA products and libraries, while the Agilent 2100 was utilized to assess the integrity of the cDNA libraries. Additionally, RT-qPCR was performed to precisely quantify the effective concentration of the libraries. Once the libraries successfully passed the inspection process, sequencing was executed using the Illumina NovaSeq 6000 platform, targeting a minimum sequencing depth of 50,000 reads per cell. The sequencing was carried out by Biomarker Technologies Corporation (performed by Biomarker Technologies Corporation, Beijing, China).

### 2.9. Processing of Single-Cell RNA-Seq Data

We processed our data using 10× Cell Ranger (v7.1.0) following these steps: First, raw sequencing data (BCL) was demultiplexed into FASTQ files with “cellranger mkfastq”. These FASTQ files were then used for comparison, filtering, and UMI counting with “cellranger count”, referencing the Gallus gallus genome (GRCg7b). Notably, mitochondrial gene annotations had to be added manually.

### 2.10. Differentially Expressed Gene (DEG) Analysis and Gene Ontology Enrichment Analysis

We processed the sequenced single cells by log-transforming expression data and filtering to retain cells expressing more than 1000 genes. Genes expressed in at least three single cells with an expression level >1 were considered. After filtering, we retained 4746 cells from the normal peritoneum group and 5053 cells from the HVP group for analysis. Using Seurat (v3.2.2), we identified the top 2000 highly variable genes for further analysis. Differential expression analysis was performed using FindAllMarkers to identify DEGs within each cluster and FindMarkers to compare two clusters. In order to realize unsupervised clustering and cell type identification, we perform the RunPCA function for dimensionality reduction in Principal Component Analysis (PCA). To determine the dimensionality of the dataset, we chose the number of PCs by the JackStraw function to obtain a data matrix that contains most of the information of the original data and found that using the first 20 PCs for dimensionality reduction clustering was the optimal choice for this experiment by calculation. First, FindNeighbors was used to calculate the nearest neighbor distance, and then Clustree, FindClusters and other functions were used to select the best resolution. It was found that when resolution >0.2, different clusters will have more entanglement under different resolutions. Then, RunUMAP and RunTSNE were used for further dimensionality reduction for clustering, and finally, the function DimPlot was used to visualize the cell clustering.

After the clustering process described above, the cell clusters underwent dimensionality reduction and were divided into unsupervised categories. The “FindMarkers” function was used to compare each cluster with all other cells (min. pct = 0.25, logfc. threshold = 0.25), enabling identification of differential marker genes. The resulting table presented the differential genes for each cluster. We first used Single R (2.2.0) for primary annotation, then employed online resources CellMarker (CellMarker (xbio. top)) and PanglaoDB (PanglaoDB—a Single-Cell Sequencing Resource for Gene Expression Data) to identify cell classes and finalize cell type annotation. See Table S5 for marker genes used in this study.

### 2.11. RNA Extraction, cDNA Synthesis, and Quantitative Real-Time PCR (RT-qPCR)

Total RNA was isolated from the peritoneum using RNAiso (Takara, Otsu, Japan) following the manufacturer’s instructions. Subsequently, reverse transcription was carried out employing HiScript Q-RT SuperMix for qPCR (+gDNA wiper) from Vazyme (Nanjing, China) to synthesize RNA into cDNA. The RT-qPCR analysis was conducted using an ABI 408 Quant Studio 5 instrument (Thermo Fisher Science, Fremont, NY, USA), and ChamQ Universal SYBR qPCR Master Mix from Vazyme (Nanjing, China) was utilized to measure the expression levels of mRNA. Data analysis of the results was performed using the 2^−ΔΔCt^ method to determine the relative gene expression. The primers used for the real-time quantitative polymerase chain reaction were designed using Premier Primer 5.0 software (PREMIER Biosoft, Palo Alto, CA, USA). Table S6 provides a list of the primers used in the quantitative polymerase chain reaction.

### 2.12. Primary Melanocyte Culture and Purification

Peritoneal tissues from 20 embryonic-aged Silkie chickens were isolated on sterile forceps using autoclaved surgical equipment, and then the peritoneum was placed in pre-warmed PBS at 37 °C and washed to remove contaminants. Then, the peritoneal tissues were completely crushed with sterile forceps. The minced peritoneal tissues were digested in a constant-temperature incubator at 37 °C for 60 min using 2U/mL Dispase II (Coolaber, Beijing, China) and 0.25% trypsin digestion solution (mixing ratio is 1:1). When the tissue fragments became flocculent, high-glucose DMEM (Gibco) containing fetal bovine serum (FBS) 10% (*v/v*) and 0.2% (penicillin/streptomycin) was added to terminate the digestion. The digested suspension was then filtered through 70 µm and 40 µm strainers and centrifuged at 1000× rpm for 10 min, respectively. The precipitated cells were seeded uniformly in cell culture dishes by blowing with melanocyte medium (Procell, Wuhan, China) within a 5% CO_2_, 37 °C incubator. When the melanocytes grew to 70–80% confluence, adherent purification was performed using 0.25% trypsin-EDTA (Gibco) for 7–8 min at 37 °C in a 5% CO_2_ incubator to eliminate the interference of other cells.

### 2.13. Dopa Staining

Melanocytes from the 5th generation, in the logarithmic growth phase, were cultured in 6-well plates at 37 °C in a 5% CO_2_ environment until they reached approximately 70% confluence. Following this, the culture medium was removed, and the cells were washed with PBS, undergoing gentle agitation for 5 min on a decolorization shaker. This process was repeated three times. After removing the PBS, a 4% paraformaldehyde cell fixative was applied and left overnight at 4 °C. Subsequently, the fixative was discarded, and the cells were subjected to three PBS washes. Upon completion of the washing steps, the cells were treated with a 3,2-dihydroxy-1-1 phenylalanine incubation solution and incubated for 30 min at 37 °C while protected from light exposure with tin foil. The initial incubation solution was replaced with a fresh batch, and the cells were further incubated at 37 °C under tin foil until the solution turned brownish black. A 0.1% Nuclear Solid Red staining solution was then applied, and staining was carried out for 10 min at room temperature. After rinsing the staining solution with distilled water, images were captured using an inverted microscope (TE2000-U; Nikon, Tokyo, Japan).

### 2.14. Immunofluorescence

When melanocytes in a 6-well plate reached 80–90% confluence, the culture medium was removed, and the cells were washed with PBS. They were gently agitated on a decolorization shaker for 5 min, repeating this step three times. The cells were then fixed with 4% paraformaldehyde overnight at 4 °C. Afterward, the fixative was removed, and the cells underwent three PBS washes. To enable permeabilization, 0.1% TritonX-100 was applied for 1 h, followed by three 5 min PBS washes. The cells were subsequently blocked with 1% BSA at 25 °C for 1 h. After aspirating the liquid, 600 μL of primary antibody (dilution ratio: MITF at 1:500) was added, and the cells were incubated for 20 h at 4 °C. The primary antibody was aspirated and removed, and the cells underwent three PBS washes. A secondary antibody was introduced, and the cells were incubated for 1 h at 25 °C, while being protected from light. Following this, the secondary antibody was aspirated and removed, and the cells were subjected to three PBS washes. Finally, Hoechst33342 staining solution was applied, and the cells were incubated in the dark for approximately 10 min. After three PBS washes, the cells were observed under an inverted fluorescence microscope (DMi8; Leica, Wetzlar, Germany).

### 2.15. Transmission Electron Microscopy

Melanocytes from the 5th generation were trypsin-digested, with digestion cessation achieved by adding complete medium and subsequent centrifugation at 1000 r/min for 10 min. Following the removal of the supernatant, cells were resuspended and mixed in complete medium. This mixture was once more subjected to centrifugation at 1000 r/min for 10 min. Melanocytes were then fixed using 2.5% glutaraldehyde for ultrastructural observation [19].

### 2.16. Cell Transfection

All transient cell transfections in this study were conducted once the cells reached 60–80% confluence, following the manufacturer’s instructions for the Lipofectamine 3000 reagent (Invitrogen). The nucleic acids were diluted using OPTI-MEM medium (Gibco). For siRNA transfection, the final concentration used was 100 nM. (si-*DCT* Sequences (5′–3′): CTCCTGTAACCAATGATCA).

### 2.17. Western Blotting

The total protein from melanocyte cells was extracted using ice-cold radioimmunoprecipitation (RIPA) lysis buffer (Beyotime, Shanghai, China) with 1 mM phenylmethyl sulfonyl fluoride (Beyotime, Shanghai, China). Proteins were separated using a 10% SDS-PAGE gel and then transferred to a polyvinylidene fluoride (PVDF) membrane (Bio-Rad, Hercules, CA, USA). After blocking for 30 min, the membrane was incubated with either anti-DCT (1:1000, Bioworld, Irving, TX, USA) or anti-GAPDH (1:2000, Bioworld, USA) at 4 °C for 12 h. The membranes were then incubated with an anti-rabbit secondary antibody (1:10,000). Chromogenic reactions were carried out using the ECL Peroxidase Color Development Kit (Beyotime, Shanghai, China), following the manufacturer’s protocol. The visualization of protein bands was performed using the Odyssey instrument (Li-cor, Lincoln, NE, USA).

### 2.18. Cell Supernatant Melanin Content Assay

Cells were prepared for the relevant experiments, and the cell supernatant was collected after 48 h for melanin content analysis. The melanin assays were conducted strictly according to the instructions provided with the Chicken Melanin ELISA Kit (MEIBIAO BIOLOGY, Shanghai, China).

### 2.19. Cell Counting Kit 8 Assay

In this experiment, 4th-generation melanocytes in the logarithmic growth phase were treated with 0.25% trypsin (Gibco). Subsequently, the cells were evenly distributed in 96-well cell culture plates and incubated at 37 °C in a 5% CO_2_ environment. When cell growth reached 60–70% confluence, they were transfected with either an overexpression plasmid or siRNA, and a blank control was established. Following transfection, 10 µL of CCK-8 reagent (Biosharp Life Sciences, Beijing, China) was added to each well, and the cells were incubated for an additional 2 h. The absorbance at 450 nm was measured using a Microplate Reader Model 680 (Bio-Rad, Berkeley, CA, USA). OD values at 450 nm were determined at 12 h, 24 h, 36 h, and 48 h after a 24 h transfection period.

### 2.20. Statistical Analysis

In this study, all results were shown as mean ± S.E.M with 3–6 independent replications. An independent sample *t*-test was used to test the statistically significant difference between groups. Chi-squared test was used to calculate significant difference for polymorphism to compare allele frequency and genotype distribution between normal peritoneum and HVP. We considered *p* < 0.05 to be statistically significant. * *p* < 0.05; ** *p* < 0.01; *** *p* < 0.001, ns: not significant.

## 3. Results

### 3.1. Identification of the Formation Causes and Composition of HVP

**Figure 1:**
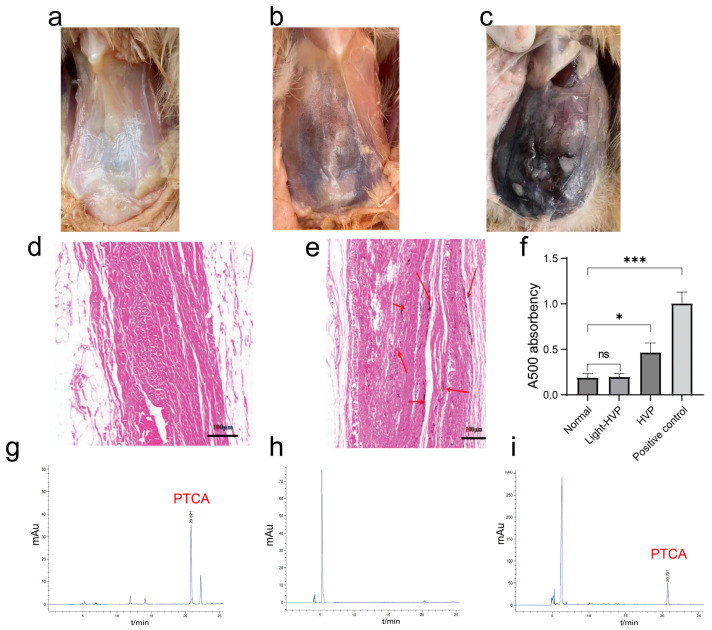
Hyperpigmentation of the visceral peritoneum is caused by abnormal accumulation of melanin. Classification of hyperpigmentation in visceral peritoneum: The degree of hyperpigmentation in the visceral peritoneum is categorized as absent (**a**), mild (**b**), or severe (**c**). Hematoxylin and eosin (HE) staining of normal peritoneum (**d**) and hyperpigmented visceral peritoneum (**e**) is depicted, with notable pigmentation within the hyperpigmented visceral peritoneal tissue highlighted by red arrows. (**f**) The results of an analysis of variance conducted subsequent to the determination of total melanin content in the peritoneum of Huiyang Bearded Chickens, using Silkie feathered ebony chickens as a positive control, are presented. (**g**–**i**) Identification of the pigment species deposited in the peritoneum of Huiyang Bearded Chickens was carried out as follows: (**g**) liquid chromatographic determination of melanin standards; (**h**) liquid chromatographic determination of normal peritoneal extracts; and (**i**) liquid chromatographic determination of peritoneal extracts exhibiting pigment deposition. Statistical significance is indicated as follows: * *p* < 0.05;; *** *p* < 0.001.

To investigate the variations in HVP formation during early growth stages, we selected individuals with identical lineage and age that were reared under standardized environmental conditions, including uniform temperature (25 ± 1 °C), humidity (60 ± 5%), lighting (16 h light/8 h dark), and a nutritionally balanced commercial diet with ad libitum access to feed and water. Anatomical examination of Huiyang Bearded Chickens revealed clear variations in visceral peritoneum pigmentation. As shown in Figure 1a–c, the degree of peritoneal pigmentation ranged from normal to progressively darker hyperpigmented phenotypes. Hematoxylin and eosin (HE) staining further confirmed the presence of irregular black deposits in the peritoneal interstitial tissue of HVP-affected chickens (Figure 1e, red arrows), which were absent in normal tissues (Figure 1d). To assess the presence of eumelanin, we followed a previously reported method [20,21] and measured the absorbance of the peritoneal samples at 500 nm using a spectrophotometer. Silkie feathered chickens were used as a positive control. The results showed a significant positive correlation between absorbance and the degree of peritoneal pigmentation (Figure 1f), with the HVP group exhibiting significantly higher absorbance values than the normal group (*p* < 0.05). High-performance liquid chromatography (HPLC) analysis was performed to determine the chemical nature of the pigmentation. The extracted pigment from HVP samples exhibited the same characteristic absorption peak at 21 min as the eumelanin standard (Figure 1g–i), confirming that the pigmentation was indeed eumelanin. A minor additional peak observed between 5 and 7 min in both normal and HVP samples was attributed to incomplete separation during processing.

Collectively, these results demonstrate that peritoneal hyperpigmentation in Huiyang Bearded Chickens is caused by excessive deposition of eumelanin.

### 3.2. RNA-Seq Analysis and Validation of Differential Gene Expression

**Figure 2:**
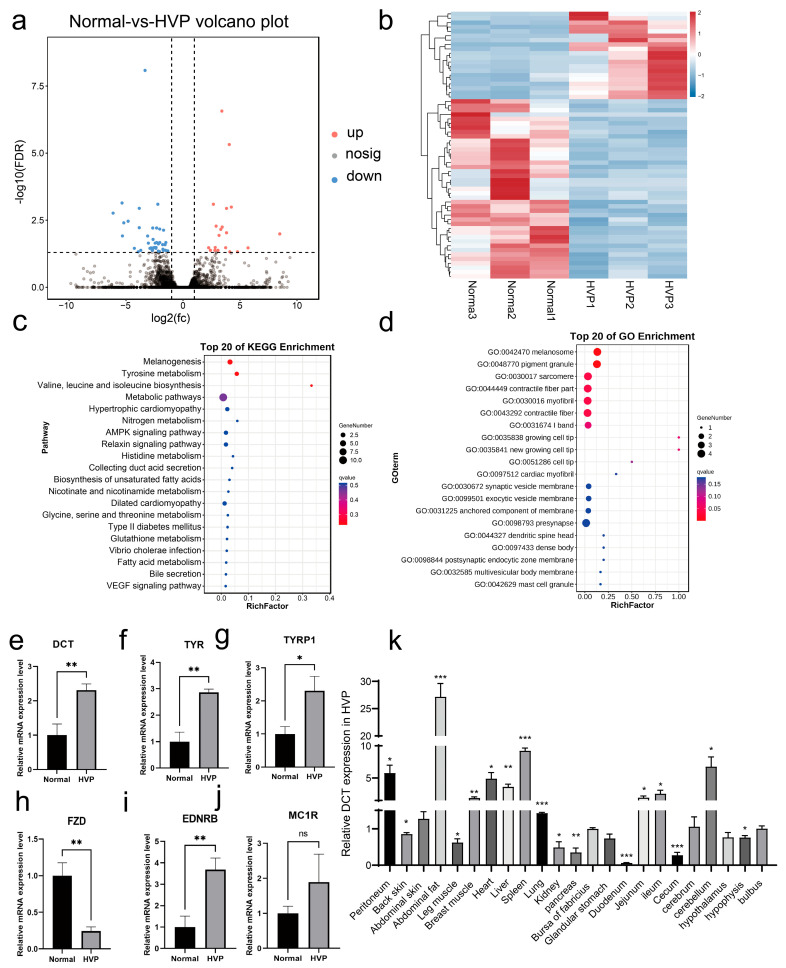
RNA sequencing reveals genes associated with HVP. (**a**,**b**) The volcano plot (**a**) and heatmap (**b**) illustrate the differentially expressed genes between hyperpigmented visceral peritoneum (HVP) and normal peritoneum in Huiyang Bearded Chickens. (**c**,**d**) The Gene Ontology functions (GO) (**c**) and Kyoto Encyclopedia of Genes and Genomes (KEGG) pathways (**d**) analyses were performed on these differentially expressed genes. (**e**–**j**) The results of quantitative reverse transcription PCR (RT-qPCR) for *DCT*, *TYR*, *TYRP1*, *FZD*, *EDNRB*, and *MC1R* genes in HVP and normal peritoneum are presented. (**k**) The tissue expression profiles of *DCT* in HVP individuals are shown. The horizontal axis represents different tissues, while the vertical axis indicates their relative expression values (mean ± S.E.M). (* *p* < 0.05; ** *p* < 0.01, *** *p* < 0.001).

To investigate the genetic regulation of hyperpigmentation in Huiyang Bearded Chickens, we classified the animals into two groups based on their peritoneal pigmentation: the normal peritoneum group and the hyperpigmented (HVP) group. A total of 17,490 genes were detected, with 61 differentially expressed genes (DEGs) identified between the two groups (Table S4). Subsequent filtration led to the identification of 20 upregulated DEGs and 41 down-regulated DEGs in HVP groups relative to the normal group across the three replicates (Figure 2a and Figure S2a). Gene expression patterns were consistent within each group but differed between groups (Figure 2b, Table S4).

Gene Ontology (GO) and Kyoto Encyclopedia of Genes and Genomes (KEGG) analyses revealed significant enrichment of biological processes and pathways related to pigmentation, such as melanogenesis, tyrosine metabolism, and metabolic pathways (Figure 2c,d). Collectively, these findings suggested potential correlations between the identified DEGs and HVP.

Expression verification of six key genes involved in melanin synthesis showed significant upregulation of *TYR*, *TYRP1*, *DCT*, and *EDNRB* in the HVP group compared to the normal group (*p* < 0.05, Figure 2e–j). Conversely, *FZD* expression was significantly reduced in the HVP group (*p* < 0.01), consistent with our transcriptomic data. Notably, the gene encoding *DCT* appears to be one of the key enzymes in eumelanin synthesis and was highly expressed in abdominal fat, spleen, cerebellum, and peritoneum (*p <* 0.05, Figure 2k).

### 3.3. Melanocyte Identification

**Figure 3:**
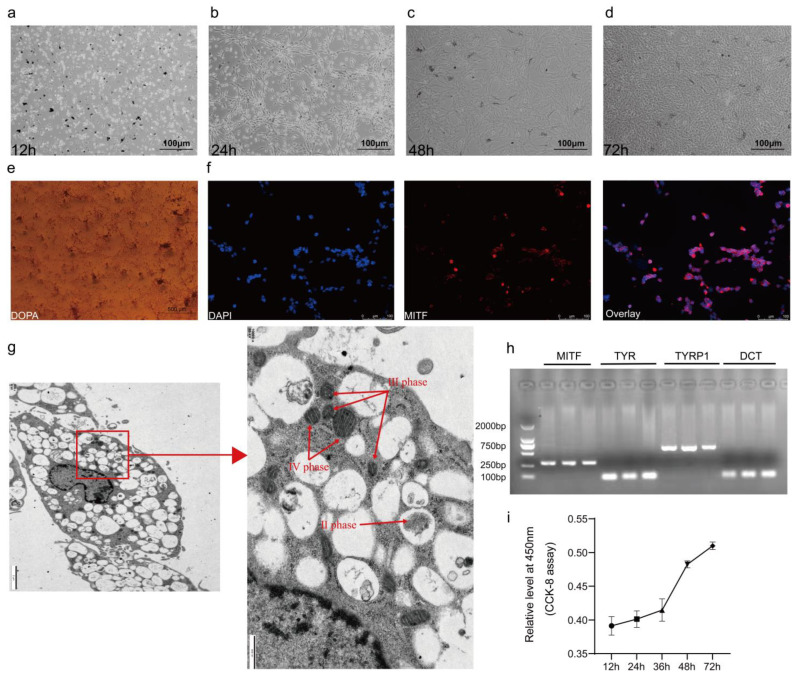
Validation of melanocyte specificity. (**a**–**d**) Depiction of the growth trajectory of melanocytes from 12 to 72 h. (**e**) Visualization of melanocytes following DOPA staining. (**f**) Results of immunofluorescence staining for MITF. (**g**) Examination of the melanocyte structure via transmission electron microscopy (2 μm), highlighting the distribution and structural features of melanosomes within melanocytes, as indicated by red arrows. (**h**) Visualization of marker genes associated with melanin production in melanocytes, namely *MITF*, *TYR*, *TYRP1*, and *DCT*, by agarose gel electrophoresis. (**i**) The CCK-8 assay results showing the growth curves of melanocytes from 12 to 72 h.

To confirm the purity of the isolated melanocytes, their growth was monitored for 72 h post-isolation (h.p.i.). At 12 h.p.i., cells exhibited multicellular characteristics, which then matured by 24 h.p.i., with melanocytes aggregating and condensing into bundles (Figure 3a,b). Significant melanocyte proliferation and the onset of melanin deposition occurred by 48 h.p.i., with substantial melanin visible in the culture medium by 72 h.p.i. (Figure 3c,d). After purification, melanocytes displayed prominent brown or black pigmentation by Dopa staining, confirming high purity (Figure 3e). Immunofluorescence assays using microphthalmia-associated transcription factor (*MITF*) confirmed melanocyte identity by detecting red fluorescence within the cells (Figure 3f). Transmission electron microscopy revealed distinct melanocyte organelles (Figure 3g, red arrows pointing). RT-qPCR of cultured melanocytes showed the expression of melanin synthesis markers, which were absent in DF-1 cells (Figure 3h and Figure S2b). Additionally, the 72 h growth curve of melanocytes demonstrated exponential growth from 36 h.p.i. (Figure 3i).

### 3.4. Functional Analysis of the DCT Gene in Melanocytes

**Figure 4:**
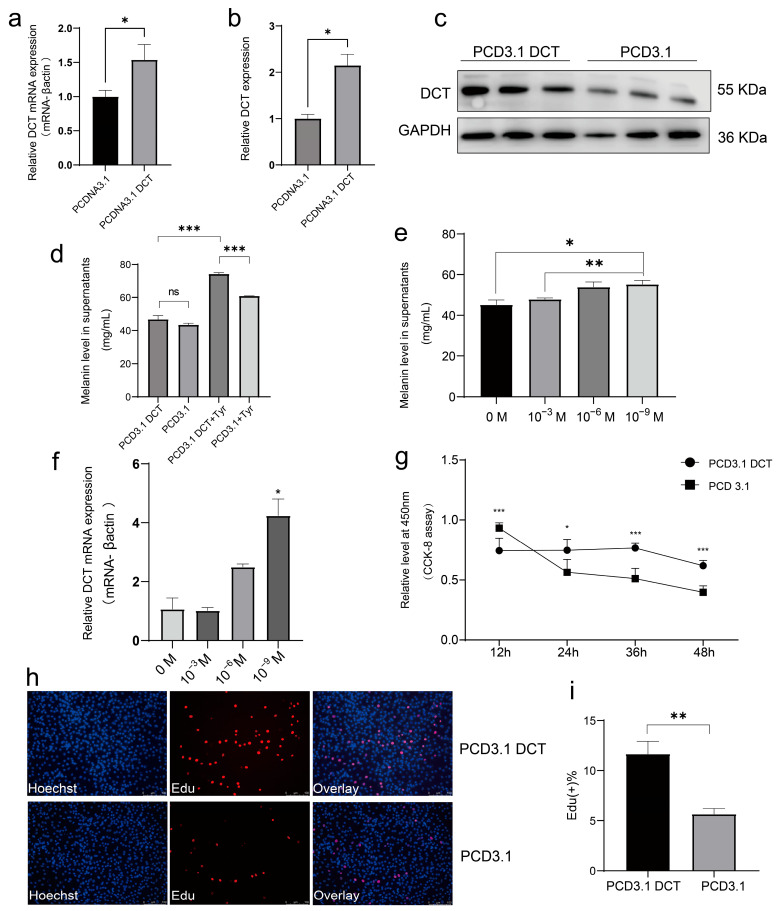
The *DCT* gene promoted melanocyte cell proliferation. (**a**) Assessment of the efficacy of *DCT* overexpression. (**b**,**c**) Western blotting analysis of *DCT* proteins following transfection with PCDNA3.1 *DCT*. Band intensities were quantified using ImageJ (1.53k) and normalized to GAPDH. Data are expressed as a fold change relative to the control. (**d**) Quantification of melanin levels in supernatants and evaluation of the impact of tyrosine addition on melanin levels in supernatant 12 h post-transfection with *DCT* in melanocytes. (**e**) Determination of melanin levels in supernatant subsequent to the addition of varying concentrations of L-tyrosine (Tyr). (**f**) Measurement of mRNA expression level of *DCT* subsequent to the addition of different concentrations of Tyr. (**g**) Representation of growth curves of melanocytes following transfection with PCDNA3.1 *DCT*. (**h**,**i**) Visualization of EdU staining (**h**) and calculation of the rate of positive EdU cells (**i**) in melanocytes post *DCT* overexpression. (* *p* < 0.05; ** *p* < 0.01; *** *p* < 0.001).

To investigate the biological role of *DCT* in melanocytes, we constructed a PCDNA3.1-DCT overexpression vector. Following transfection, *DCT* mRNA expression was significantly increased (*p* < 0.05, Figure 4a), and *DCT* protein levels were confirmed by Western blotting (*p* < 0.01, Figure 4b,c). However, overexpression of *DCT* did not result in any significant alteration in the melanin content of the supernatant in melanocytes (Figure 4d). We hypothesized that this lack of effect could be due to the limited availability of L-tyrosine, a precursor for melanin biosynthesis. To test this, we supplemented the culture with exogenous L-tyrosine (L-Tyr, 10^−9^ M), which significantly increased melanin content (*p* < 0.05, Figure 4e). Additionally, *DCT* overexpression combined with L-Tyr supplementation further enhanced melanin levels in the supernatant (Figure 4e). RT-qPCR confirmed that *DCT* expression was significantly upregulated following tyrosine supplementation (Figure 4f). Furthermore, CCK-8 and EdU staining assays showed that *DCT* overexpression promoted melanocyte proliferation (Figure 4h,i).

**Figure 5:**
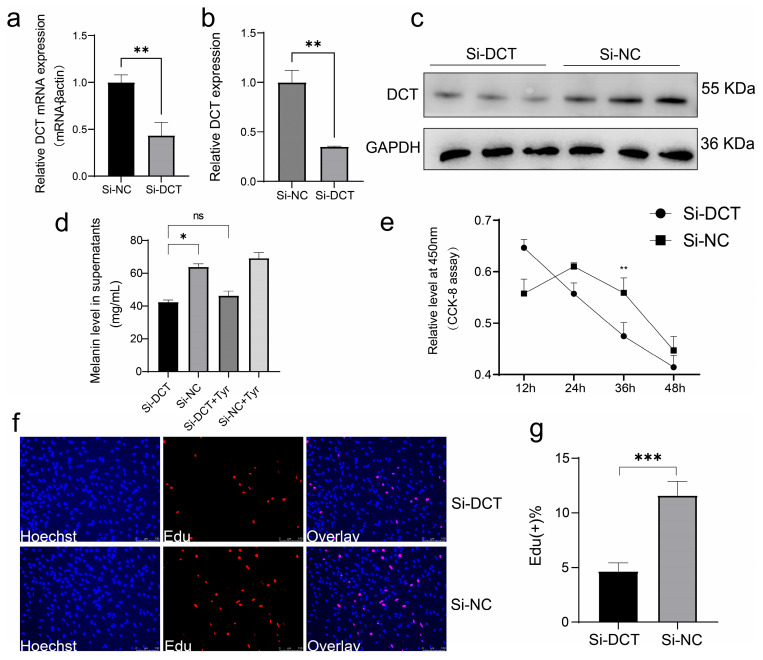
Interference with the *DCT* gene expression decreases melanin production. (**a**) Quantification of *DCT* mRNA levels in melanocytes post-transfection with the *DCT* silencing vector. (**b**,**c**) Western blotting analysis of *DCT* proteins following transfection with Si-DCT. (**d**) Evaluation of the impact of tyrosine addition on the melanin levels in supernatant 12 h post-transfection of Si-DCT in melanocytes. (**e**) Presentation of growth curves of melanocytes following transfection with PCDNA3.1 DCT. (**f**,**g**) Visualization of EdU staining (**f**) and determination of the rate of positive EdU cells (**g**) in melanocytes after *DCT* silencing. (* *p* < 0.05; ** *p* < 0.01, *** *p* < 0.001).

To validate our hypothesis, we employed siRNA to interfere with *DCT* gene transcription (Figure 5). Initially, we assessed the efficiency of this interference approach through RT-qPCR and Western blotting (Figure 5a–c). Both mRNA and the protein expression level of *DCT* were significantly reduced following *DCT* silencing (*p* < 0.01). Subsequent analysis of melanin in the cell supernatant revealed a marked decrease in melanin secretion post-silencing of the *DCT* gene (Figure 5d). Moreover, exogenous addition of L-Tyr after si-*DCT* transfection led to a significant increase in melanin synthesis. However, in the absence of functional *DCT* gene expression, the additional supplementation of L-Tyr remained ineffective, highlighting the crucial role of *DCT* in modulating melanin secretion by melanocytes. Additionally, CCK-8 assessment assays and Edu staining assays confirmed that silencing *DCT* suppressed melanocyte proliferation (*p* < 0.01, Figure 5e–g).

### 3.5. Single-Cell Transcriptome Sequencing Analysis of Peritoneal Tissue

**Figure 6:**
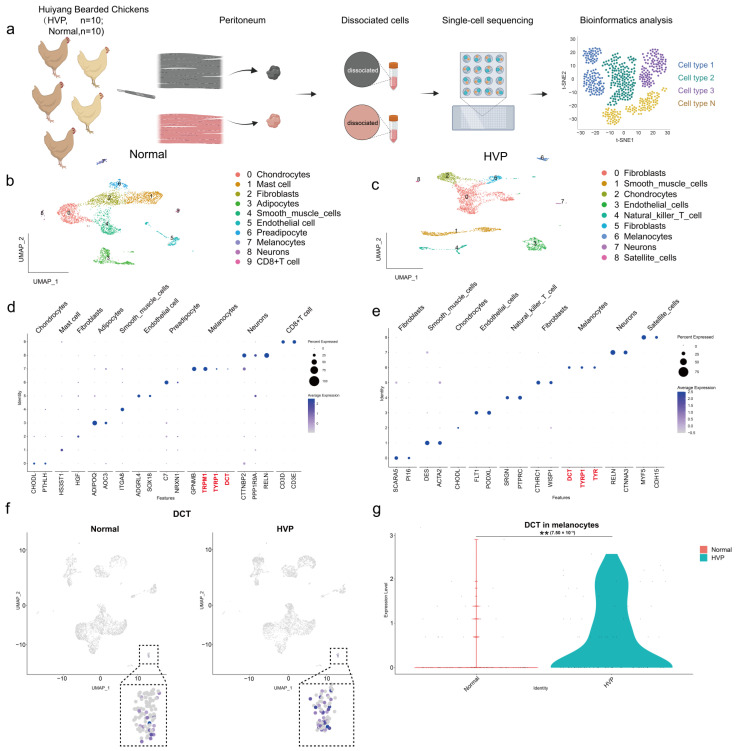
Overview of the single-cell landscape for HVP and normal peritoneum. (**a**) Presentation of the single-cell profiling design workflow. (**b**,**c**) UMAP visualization of total cells acquired from 10 normal peritoneum (**b**) and 10 HVP (**c**), with color-coded assignments based on cell type. (**d**,**e**) Dot plot depicting the expression of cell types in normal peritoneum (**d**) and HVP (**e**) cell types, where dot size and color denote the percentage of the marker gene. UMAP visualization (**f**) and violin plots (**g**) demonstrating the *DCT* expression level in HVP and normal groups. (** *p* < 0.01).

To investigate cellular heterogeneity and the molecular profile of melanocytes in normal and HVP, we performed single-cell transcriptomics (Figure 6a). After rigorous cell filtration, we obtained 4746 high-quality cells from the normal peritoneum group and 5053 cells from the HVP group (Figures S3–S5). Unsupervised clustering analysis and UMAP identified 10 distinct cell types in the normal group and 9 in the HVP group, with melanocytes identified by the *DCT* gene in both groups and fibroblasts being the predominant cell type (Figure 6b–e). Focusing on *DCT* gene expression, we identified melanocyte clusters (cluster 7 for the normal group and cluster 6 for the HVP group), with 93 melanocytes from the normal group and 76 melanocytes from the HVP group (Figure 6f). Violin plot analysis of the entire cell landscape revealed a significant increase (*p* < 0.01) in *DCT* expression abundance in the HVP group compared to the normal group (Figure 6g).

**Figure 7:**
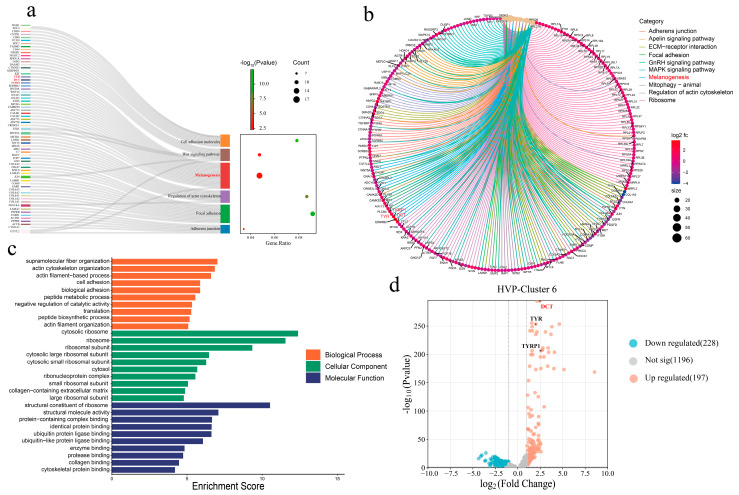
Overview of the HVP single-cell sequencing enrichment analysis. (**a**) KEGG pathways analysis of differentially expressed genes between HVP and normal peritoneum. GO functions (**b**) and KEGG pathways (**c**) analysis of HVP-cluster 6 differentially expressed genes. (**d**) Volcano plot showing differentially expressed genes in HVP-cluster 6.

KEGG analysis of differentially expressed genes (DEGs) in the HVP group implicated their involvement in signaling pathways related to melanin production, such as melanogenesis and the Wnt signaling pathway (Figure 7a). Additionally, KEGG and GO terms for DEGs within cluster 6 (i.e., melanocytes) of the HVP group suggested their impact on the melanogenesis pathway, ECM–receptor interaction, and the GnRH signaling pathway (Figure 7b,c). Finally, we quantified the expression level of genes involved in melanogenesis (i.e., *DCT*, *TYR*, and *TYRP1*), revealing an approximate 2.5-fold increase in expression (Figure 7d). Collectively, these analyses corroborated that the characteristic of HVP is attributable to the upregulation of *DCT*, *TYR*, and *TYRP1* genes.

## 4. Discussion

HVP presents a persistent challenge in the selective breeding of Chinese indigenous chicken breeds, particularly the bearded chicken. Recent investigations have demonstrated that HVP has not been reported in white-feathered broilers but is predominantly observed in colored-feathered chicken breeds [2]. This distinct distribution pattern may be attributed to the inhibition of melanogenesis and melanocyte migration in white-feathered chickens, which is regulated by recessive or dominant white alleles [22]. A classic example involves the insertion of an endogenous avian retrovirus into the TYR gene, resulting in the recessive white phenotype that blocks TYR expression and melanin biosynthesis [23,24,25]. Similarly, mutations in the premelanosome protein (PMEL) gene at the dominant white locus alter melanosome morphology, thereby disrupting pigment deposition [26,27,28]. These genetic mechanisms provide plausible explanations for the absence of HVP in white-feathered commercial broilers.

Interestingly, the HVP phenotype has also been observed in birds affected by runting and stunting syndrome (RSS). Previous genetic studies have reported a moderate negative genetic correlation between HVP and immune traits, particularly the antibody response to Newcastle disease virus (rg = −0.42). In addition, HVP also leads to a relatively high ratio of heterophil to lymphocyte (H/L) in chickens [1], indicating that chickens exhibiting HVP may have compromised immune resilience [29,30]. Additionally, the prevalence of HVP appears to increase under suboptimal farming conditions [19], suggesting that environmental stressors may exacerbate the manifestation of HVP. These findings imply that HVP may serve not only as a pigmentation abnormality but also as an indirect indicator of reduced immune competence and heightened environmental sensitivity.

The relationship between HVP and growth traits remains controversial. Early research by Luo et al. (2013) reported a positive genetic correlation between HVP and body weight at 91 days of age (*p* < 0.05), with heritability estimates suggesting favorable associations with carcass traits such as body weight (rg = 0.27), carcass weight (rg = 0.24), net weight (rg = 0.27), breast muscle weight (rg = 0.17), and leg muscle weight (rg = 0.34) [1]. In contrast, more recent findings from the same research team revealed that HVP exerts a negative impact on growth performance, with affected chickens displaying significantly reduced body weights at 21, 42, 70, and 91 days (*p* < 0.05) [19]. These contradictory results highlight the complex genetic and physiological underpinnings of HVP and underscore the need for further mechanistic investigations.

In the present study, we sought to elucidate the genetic mechanism and molecular regulation of HVP. Although several candidate genes related to visceral pigmentation have been proposed in previous studies, most lacked rigorous functional validation [2,3,14,18,31,32]. Here, using spectrophotometry and HPLC, we confirmed that the black deposits in the peritoneum of HVP-affected chickens consist of eumelanin, consistent with previous studies employing infrared spectroscopy [14]. To further dissect the molecular underpinnings of HVP, we employed an integrative multi-omics approach combining bulk RNA sequencing, single-cell transcriptomics, and molecular validation assays. Our analyses revealed that key melanogenesis-related genes, including *DCT*, *TYR*, and *TYRP1*, were significantly upregulated in the HVP-affected chickens. These transcriptomic results were further corroborated by previously published Data-Independent Acquisition (DIA) proteomics datasets from the 40-day-old peritoneum of Huiyang Bearded Chickens, which identified overlapping differentially abundant proteins involved in melanogenesis and tyrosine metabolism [3]. Enrichment analyses of GO terms and KEGG pathways consistently revealed significant involvement of melanogenesis and tyrosine metabolism pathways in both transcriptomic and proteomic datasets. Furthermore, single-cell transcriptomics allowed us to construct a high-resolution cellular atlas of the peritoneum and reveal consistent pathway enrichment within melanocyte-specific clusters (i.e., HVP-cluster 6) [33,34,35], notably in melanogenesis, wingless/integrated (Wnt) signaling pathway, extracellular matrix (ECM)–receptor interactions, and gonadotropin-releasing hormone (GnRH) signaling. These findings align with recent observations by Chen et al. [3], who underscored the role of Wnt signaling in neural crest cell (NCC) migration—a critical developmental process influencing melanocyte distribution and pigmentation patterns. Given that visceral melanocytes are derived from NCCs, it is plausible that upstream developmental pathways such as Wnt and retinoic acid signaling regulate melanocyte migration, differentiation, and melanin deposition in the peritoneum. The integration of our transcriptomic data with these developmental insights provides a comprehensive model for understanding the pathogenesis of HVP, bridging molecular melanogenesis with embryonic developmental processes.

Melanin, primarily synthesized by melanocytes, exists as either eumelanin or pheomelanin and is responsible for pigmentation in vertebrates [6,7]. While the genetics and physiology of melanin biosynthesis have been extensively studied in mammals, its regulation at the single-cell level in avian internal tissues remains poorly characterized [36,37,38]. Our findings demonstrate that *DCT* plays a central role in controlling eumelanin synthesis. *DCT* catalyzes the conversion of dopachrome to DHICA, reducing the accumulation of cytotoxic intermediates such as DHI and DOPA [39]. These intermediates, if unregulated, can trigger oxidative stress and impair melanocyte viability [40,41]. Our functional validation further supports the key regulatory role of *DCT* in melanogenesis. While *DCT* overexpression alone did not significantly elevate melanin content, supplementation with exogenous L-Tyr significantly enhanced melanin production at a concentration of 10^−9^ M, consistent with the established dependency of melanogenesis on tyrosine availability [42]. Conversely, siRNA-mediated silencing of *DCT* significantly reduced melanin secretion and suppressed melanocyte proliferation, likely due to cytotoxic accumulation of intermediate metabolites. These results mirror findings from mammalian models where *DCT* deficiency impairs melanocyte function and increases susceptibility to oxidative damage [36,40,43,44,45].

To our knowledge, this is the first study to utilize *DCT* as a molecular marker for identifying melanocytes in avian peritoneal tissues. These findings not only enhance our understanding of melanogenesis in non-dermal avian tissues but also provide practical molecular targets for breeding programs aimed at reducing the incidence of HVP, improving carcass appearance, and minimizing economic losses in poultry production. Future research exploring gene-editing approaches, such as CRISPR/Cas9-mediated *DCT* modulation, and further investigation into the upstream developmental pathways governing melanocyte migration and differentiation could offer promising avenues for the management of this economically significant trait.

### Limitations

This study has several limitations. First, the relatively small sample size for transcriptomic and functional analyses may restrict the generalizability of our findings across chicken populations. Second, the single-cell transcriptomic results may have been affected by limited melanocyte viability and yield from HVP tissues, potentially explaining the lack of observed increase in melanocyte abundance. Finally, as this work focused mainly on molecular mechanisms, environmental and nutritional factors influencing HVP were not investigated. Future studies integrating genetic, environmental, and management factors will help provide a more comprehensive understanding of HVP development.

## 5. Conclusions

In conclusion, our study sheds light on the underlying molecular mechanisms behind HVP in chickens, which have significant implications for both animal health and the appearance of commercial chicken carcasses. Through a comprehensive multi-omics approach and experimental validations, we have identified melanin accumulation as the primary driver of HVP, originating from increased expression of the *DCT* gene within melanocytes. SNP screening suggests *DCT* gene polymorphism (g.147917398 C > T) could serve as a genomic marker for improving chicken carcass quality through selective breeding. By elucidating the genetic basis of HVP, our research contributes to the development of targeted strategies for mitigating this condition in poultry production, ultimately benefiting both producers and consumers alike.

## Data Availability

The RNA-Seq datasets generated and/or analyzed during the current study are available in the China National GeneBank DataBase (CNGBdb) repository under the accession number CNP0004933. The scRNA-Seq data generated in this study are available at NCBI GEO under the following accession numbers (GEO: GSE246996). Any remaining data could be acquired in the supplement files or requested from the corresponding author.

## Supplementary Materials

The following supporting information can be downloaded at https://www.mdpi.com/article/10.3390/ani15213076/s1, Figure S1: The information on the SNP in *DCT*; Figure S2: (a) Differentially expressed genes identified in HVP relative to the normal peritoneum of chicken using RNA-seq. (b) The marker genes associated with melanin production of *MITF*, *TYR*, *TYRP1* and *DCT* could not be amplified in DF-1 cells visualized by agarose gel electrophoresis; Figure S3: Procedure of single-cell sequencing data analysis in the normal peritoneum group; Figure S4: Procedure of single-cell sequencing data analysis in the HVP group; Figure S5: UMAP visualization of double cell distribution after quality control of single-cell sequencing results; Figure S6: Different display of single-cell sequencing marker genes; Table S1: The information on the SNP in *DCT* in this study; Table S2: Genotype, allele frequency, and diversity parameters of SNP in *DCT*; Table S3: Chi-squared test analysis of SNP on the *DCT* gene with chicken peritoneum traits; Table S4: The differentially expressed genes in the HVP group relative to the normal peritoneum of RNA-seq; Table S5: Marker genes used for cell type annotation; Table S6: Primers sequences used in this study.

## Abbreviations

HVP: hyperpigmentation of the visceral peritoneum; HE: hematoxylin and eosin staining; GEMs: gel beads in the emulsion; PCA: Principal Component Analysis; UMIs: unique molecular identifiers; DEPC: diethyl pyrocarbonate; HPLC: High-Performance Liquid Chromatography; HB: Huiyang Bearded Chicken.
