# Supplementary material for: Visceral Peritoneum Hyperpigmentation in Chickens Is Associated with DCT Expression
Source: Animals (Basel). 2025 Oct 23;15(21):3076. doi: 10.3390/ani15213076 (PMC12607967; doi:10.3390/ani15213076)
Supplement: Supplementary file 1 [file animals-15-03076-s001.zip › supplementary file.pdf]

Supplemental information for "

Visceral Peritoneum Hyperpigmentation in Chickens Is Associated with DCT Expression "

Supplemental result

The correlation analysis of polymorphism of DCT gene on HVP traits.

The *DCT* gene of 347 individuals was PCR-amplified and sequenced to identify the potential site carrying the mutation by Sanger sequencing and genotyped by DNASTAR (Figure S1a-b). We identified one significant SNP (Single nucleotide polymorphism) site (i.e., NC_006088.5: g.147917398 C>T), which is a synonymous mutation located in exon region of *DCT* gene (Table S1). The allele that matched the NCBI reference sequence (GRCg6a, NC_006088.5) was defined as wild type. Otherwise, the allele was defined as mutant type.

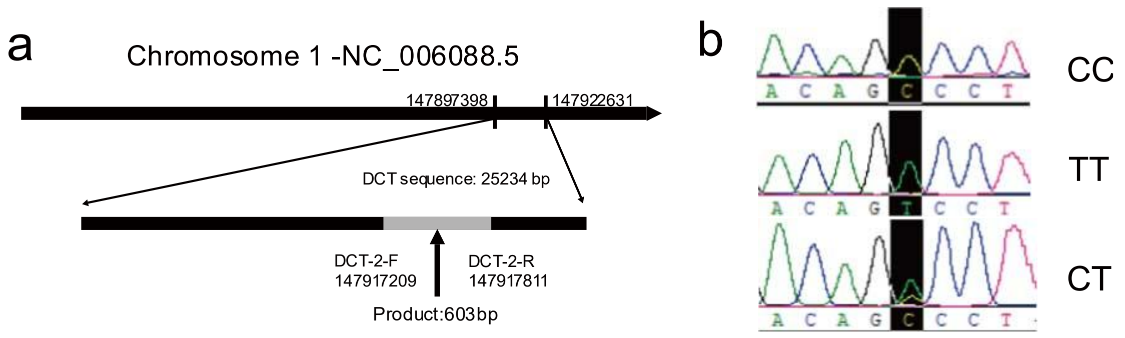

Figure S1 The information on SNP in *DCT*. (a) Schematic representation of the SNP site. (b) Sanger sequencing of the SNP loci of the DCT gene were identified in this study.

Table S1. The information on SNP in *DCT* in this study.

Gene	SNP ID	Location	Amino change	Mutant type	Heterozygote	Wild type
DCT	NC_006088.5: g.147917398 C>T	Exon 6	NO	TT (n=222)	CT (n=108)	CC (n=17)

After genotyping, the genotype frequencies, allele frequencies and diversity parameters of SNP site in *DCT* were calculated and listed (Table S2). The calculation result shows that the SNP site in DCT was consistent with Hardy-Weinberg equilibrium (HWE, p > 0.05). At the SNP site, the wild allele "T" (0.795) was more frequent than the mutant allele "C" (0.205). The genotype frequency homozygous TT had the highest genotype frequency than heterozygous CT and homozygous CC. In addition, the polymorphism information content (PIC) value was moderate polymorphism (PIC=0.2723,0.25 < PIC < 0.5), suggesting the genetic diversity of the identified DCT SNP site.

Table S2. Genotype, allele frequency, and diversity parameters of SNP in *DCT*.

Gene	SNP ID	Genotype frequency			Allele frequency			Population parameters			
DCT	NC_006088.5: g.147917398 C>T	TT	CT	CC	T	C	P_{HWE}	Ho	He	Ne	PIC
		0.640	0.311	0.049	0.795	0.205	0.415	0.675	0.325	1.483	0.273

Note: HWE, Hardy-Weinberg equilibrium; Ho, homozygosity; He, heterozygosity; Ne, effective allele numbers; PIC, polymorphism information content.

Next, to explore the relationship between the obtained SNP site and HVP traits, chi-

squared test analysis of SNP on *DCT* gene was performed (Table S3). In *DCT*, the SNP site was significantly associated with HVP traits ($P < 0.05$). Thus, these indicated that the SNP of DCT can be a potential molecular marker for the traits of HVP.

Table S3. Chi-squared test analysis of SNP on DCT gene with chicken Peritoneum traits.

SNP ID	Peritoneum traits		Genotype			Total	$\chi 2$	P-Values
			TT	CT	CC	162		
	Normal		(46.4%)	(42.6%)	(76.5%)	(46.7%)		
NC_006088.5: g.147917398 C>T							6.811	0.033
			TT	CT	CC	185		
	HVP		(53.6%)	(57.4%)	(23.5%)	(53.3%)		
		Total	222	108	17	347		

Note: $\chi 2$, chi-square test.

Supplemental information

Table.S4 The differentially expressed gene in HVP group relative to normal peritoneum of RNA-seq. (refer to additional Table S4)

Table.S5 Marker genes used for cell type annotation. (refer to additional Table S5)

Table.S6 Primers Sequences used in this study.

Gene Name	Primer Sequences (5’–3’)	Application
DCT-1	F: CCACCAGTTGTCAGGAAG<br>R: GGACCAAGCAGACTCATC	PCR
DCT-2	F: GCTTCAACCCTTTCTCCTAA<br>R: GGTGTGGCAGAGTTCAAG	Genotyping
MITF	F: GGAACTTGAGAACAGACAGA<br>R: CCAGGAGACACAGAGGAA	PCR
TYR	F: CAGGAGTGGATGACAGAGA<br>R: TAGGTTAAGGTAGGCAAGGA	PCR
TYRP1	F: GATGAGTGGCTAAGAAGGTAT<br>R: TGGCTTGGTGTTGCTATG	PCR
q-*DCT*	F: GGCAATCCAACAGCAGATTAAC<br>R: TCAGGGTGTTCCATAGTTTCCA	RT-qPCR
q- *MITF*	F: TGCTGGTGCTCTACATTC<br>R: ATGAGGTGGAAGAAGAAGG	RT-qPCR
q-*TYR*	F: TCCTTGCCTACCTTAACCTA<br>R: ACACATTGGAGCCACCTA	RT-qPCR
q-*TYRP1*	F: CCAAGCCAAGGTGACAAT<br>R: CCTGACGGAATAATAATGAGA	RT-qPCR
q-*FZD*	F: TCTGCCTGTATGTTGTTGT<br>R: GCTTGTCCTGGTTCTCTT	RT-qPCR
q-*EDNRB*	F: GTGTGAAGCCAGCAGATAT<br>R: CAATGAGGATGTAGAGAAGGT	RT-qPCR
GAPDH	F: GGTGAAAGTCGGAGTCAACGG<br>R: TCGATGAAGGGATCATTGATGGC	RT-qPCR
β-*actin*	F: GATATTGCTGCGCTCGTTG<br>R: TTCAGGGTCAGGATACCTCTTT	RT-qPCR

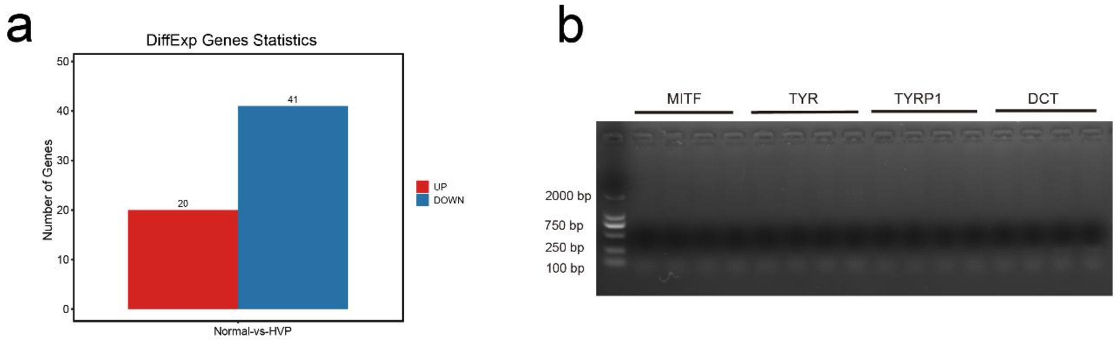

Figure S2

(a) Differentiated-expressed genes identified in HVP relative to normal peritoneum of chicken by using RNA-seq. (b) The marker genes associated with melanin production of *MITF*, *TYR*, *TYRP1* and *DCT* could not be amplified in DF-1 cell visualized by agarose gel electrophoresis.

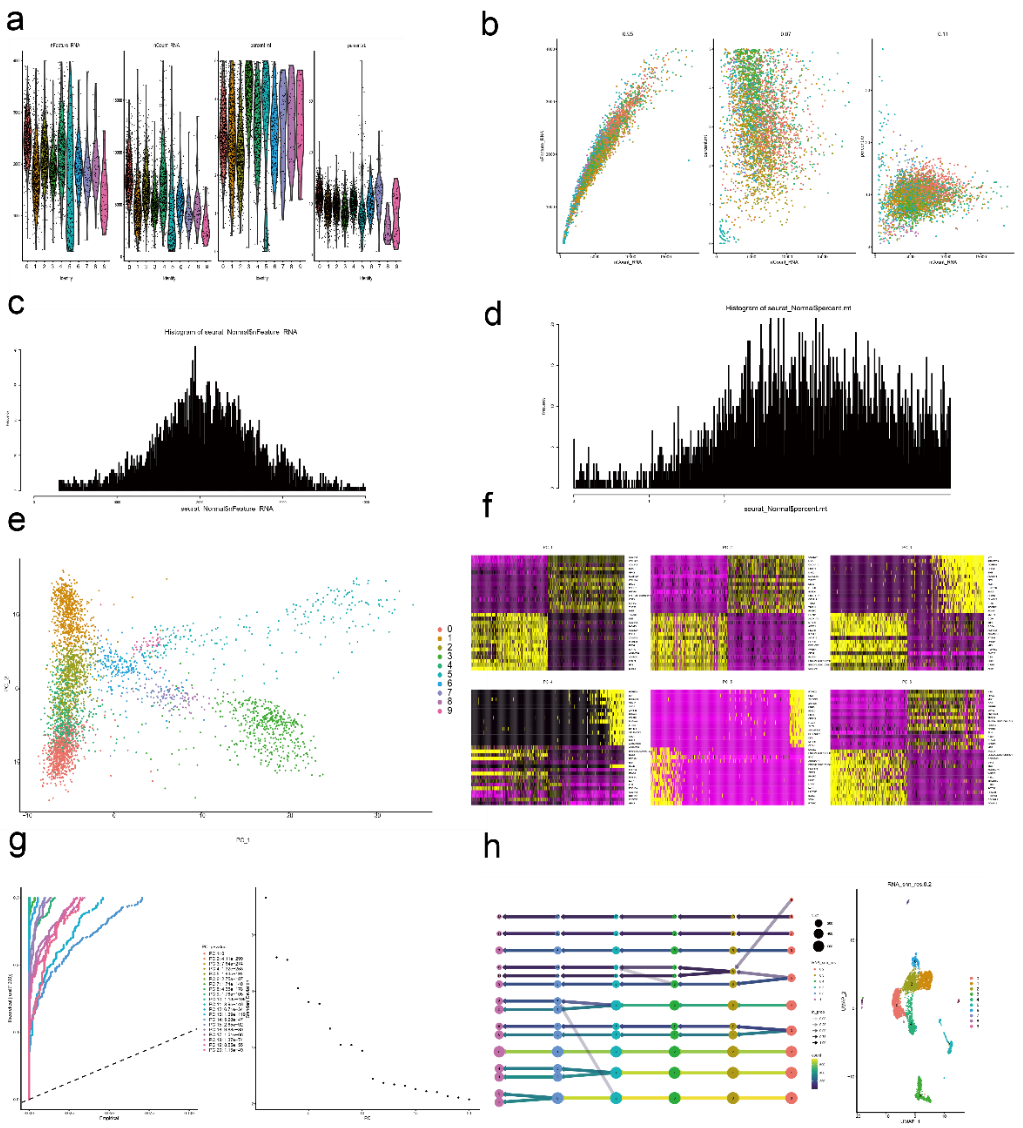

Figure S3 Procedure of single cell sequencing data analysis in normal peritoneum group.

(a) Quality control metric for normal peritoneum of single-cell sequencing. (nFeature_RNA: the number of genes in cells; nCount_RNA: the number of UMIs in cells; percent.mt: the distribution ratio of mitochondrial gene content in cells; percent.rb: the distribution ratio of ribosome gene content in cells). (b) Scatter plot of correlation between nCount_RNA and nFeature, percent.mt, percent.rb. (c) Histogram of nFeature_RNA after quality control. (d) Histogram of percent.mt after quality control.

(e) Clustering of normal peritoneum cells following Principal component analysis (PCA). (f) A heat map of the expression of gene composition in the first 6 PCs following PCA. (g) p values of the top 20 PCs determined by PCA and variance of different PCs. (h) Find the optimal resolution.

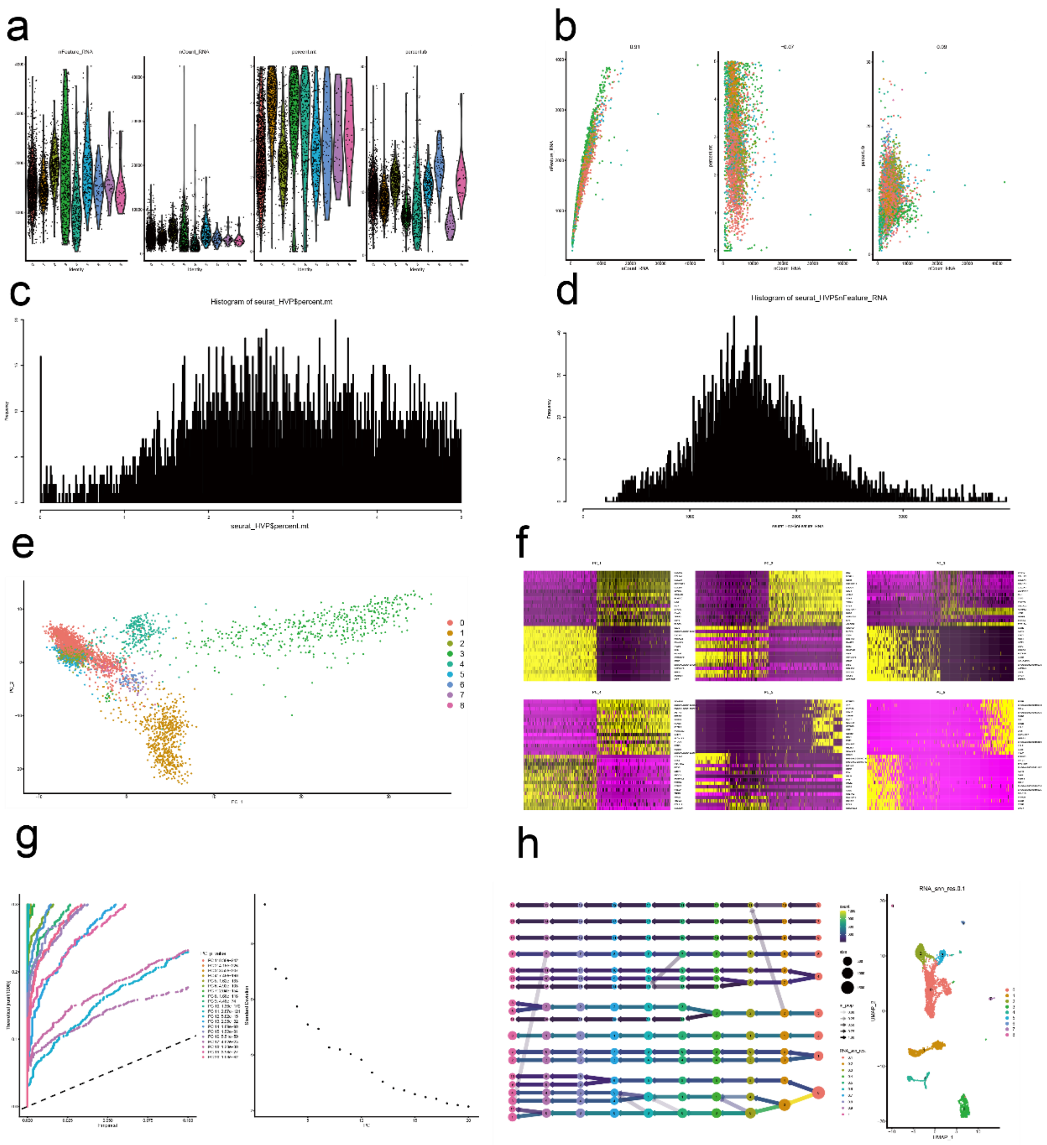

Figure S4 Procedure of single cell sequencing data analysis in HVP group.

(a) Quality control metric for HVP of single-cell sequencing. (nFeature_RNA: the number of genes in cells; nCount_RNA: the number of UMIs in cells; percent.mt: the

distribution ratio of mitochondrial gene content in cells; percent.rb: the distribution ratio of ribosome gene content in cells). (b) Scatter plot of correlation between nCount_RNA and nFeature, percent.mt, percent.rb. (c) Histogram of nFeature_RNA after quality control. (d) Histogram of percent.mt after quality control. (e) Clustering of HVP cells following PCA. (f) A heat map of the expression of gene composition in the first 6 PCs following PCA. (g) p values of the top 20 PCs determined by PCA and variance of different PCs. (h) Find the optimal resolution.

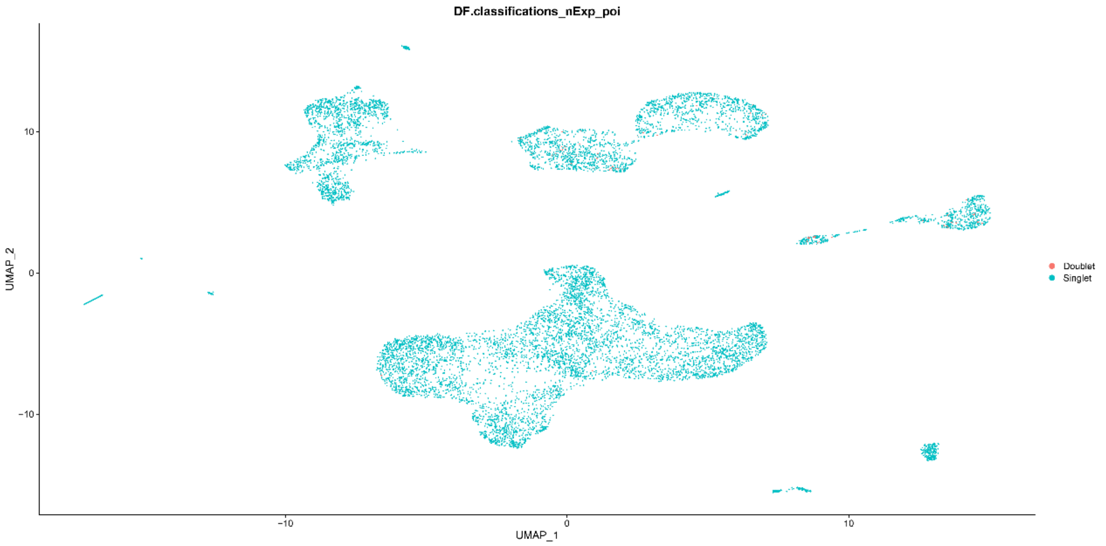

Figure S5 UMAP visualization of double cell distribution after quality control of single cell sequencing results.

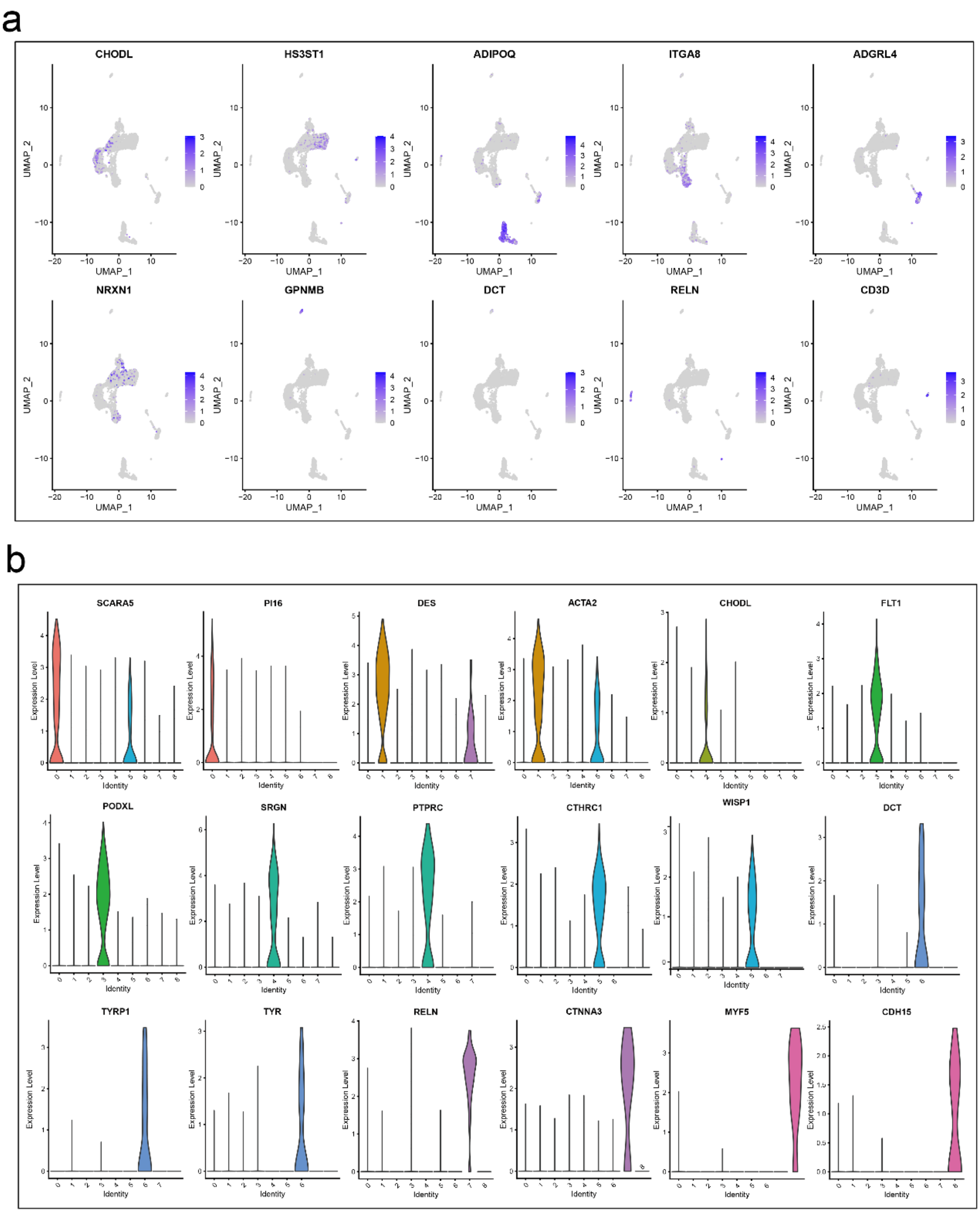

Figure S6 Different display of single-cell sequencing marker genes.

UMAP visualization (a) in normal group cell types and violin plots (b) of the marker genes expression in HVP cell types (depth of purple in(a) indicates expression level).
